# Supplementary material for: Ten simple rules for making a vocabulary FAIR
Source: PLoS Comput Biol. 2021 Jun 16;17(6):e1009041. doi: 10.1371/journal.pcbi.1009041 (PMC8238180; doi:10.1371/journal.pcbi.1009041)
Supplement: Attachment — Submitted filename: CoxEtAl10RulesVocabularySMR.pdf [file pcbi.1009041.s001.pdf]

Review of Ten Simple Rules for making a vocabulary FAIR

Simon J D Cox, Alejandra N Gonzalez-Beltran, Barbara Magagna, Maria-Cristina Marinescu

The intention of this paper is a useful contribution to address an important problem for implementation of the Semantic Web, but I think the text needs some work. Recommendation is to accept with revisions.

The pdf file contains many comments. Some others are here, followed by some conceptual suggestions.

Comments

There are various requirements that are mixed in with the rules. I think the rules could be refactored to better encapsulate the requirements (see suggestion in the conceptual section, below).

The text uses a some terminology that might not be clear to users, e.g.

annotation (e.g. line 27, 62 80) "An annotation is extra information associated with a particular point in a document or other piece of information." (https://en.wikipedia.org/wiki/Annotation). maybe -- annotate: "specify the kinds of entities in data or metadata schema, or the values of categorical properties in data or metadata"

code e.g. (line 61)

IRI—in general I think this could be replaced by 'identifier', 'web identifier', or 'resolvable identifier' most places its used.

The text assumes familiarity with web architecture. Is it clear that the readers will have this background? Examples: *'web resource'* (line 56), *web-standard* (line 76), *'on the web'* line 95, *'web identifier'* line 96. If not there should be some background on web architecture and semantic web, at least as a link to a quick overview, covering topics like these:

- URI is a name designed to be unique, there are many schemes. A URI is a name that can be encoded using standard Latin alphabet, numbers and a limited set of other characters (the ASCII character set), following rules specified in IETF RFC (https://tools.ietf.org/html/rfc3986)
- IRI is a generalized URI that allows names constructed with non-Latin characters and a broader set of symbols (anything that can be encoded using UTF-8)
- The World Wide Web (the Web) is a technology for associating identifiers with locations in a global network of computers (the Internet) and retrieving files (resources) from those locations. Retrieved resources can contain resolvable identifiers for other resources, thus creating a web of linked documents.
- A resolvable identifier is an identifier for which there is a known procedure to determine what it identifies and how to obtain a representation of that thing. ('resolve' might be less alien to users that 'dereference')

- An HTTP URI (or IRI) is a specific kind of identifier that is resolvable using the World Wide Web technology.
- On the Web, an identified thing is a resource; that resource might have multiple representations. A representation is an artifact (digital document on the web) intended to convey information about some resource(a thing);

Comments on table following line 203

- "Encode each vocabulary term as an owl:Class, or as an instance of an owl:Class if it is the most specific concept in the vocabulary [16]. The term identifier should be assigned as discussed in Rule 5"
- problematic-- why are leafs instances? seems inconsistent, terms in vocab should all be instances or all be classes. Need examples
- Can't use equivalent class for synonyms if term is an instance.
- "Synonyms can be encoded as annotations" ? examples? what annotation vocabulary?
- "Add codes and symbols" have to explain what you mean by 'code'
- Multiple rdfs:label in owl is tricky (unless different languages...)-- client randomly picks one?
- Define the hierarchy of terms: in owl, carefully evaluate nature of hierarchy, is it kindOf (use subclass), or partOf, ..

Conceptual suggestions

A clearer statement of what FAIR means for a vocabulary would be useful. Something like:

FAIR in the context of a vocabulary:

- Find -- search for term and find representation that binds a text label (symbol) with intended meaning (via a text definition), and a resolvable identifier; given an identifier, get a representation to explain what it means; provide metadata documenting provenance.
- accessible-- there is an online mechanism by which given the identifier, a useful representation can be obtained that conveys the meaning and provenance of a vocabulary item; the entire vocabulary can be accessed as a unit online.
- interoperable-- at least one accessible representation conforms to a recognized scheme for serializing information about the vocabulary item
- reusable-- license; sufficient metadata to trust; definitions of terms are sufficient to understand what they mean.

Assumptions-- necessary condition for a vocabulary is a set of unique labels. Depending on the nature of the vocabulary, those labels might or might not be bound to unique meaning. There should be a recommendation to specify if identifire is bound to the meaning, or to the label.

Possible refactoring of rules:

Vocabulary management and stewardship

Rule 1. Verify that the legacy-vocabulary license allows repurposing

Rule 2. Determine the governance arrangements and custodian responsible for the legacy vocabulary

Rule 3 (new) Determine the license that will be assigned to the online vocabulary

Rule 4 (was Rule 10). Implement a process for maintaining the vocabulary content and online access

engage with the community for whom the vocabulary is provided, through the subject matter experts that created the vocabulary

Vocabulary content

Rule 5 (was Rule 3). Check minimal term definition completeness

- Check that labels are unique
- Have reconcile this with the

Rule 6 (was Rule 7). Add rich metadata –

- Define the scope and purpose of the vocabulary
- provenance and ownership information (citation of or links to the source, pointers to the organization or community responsible for the content),
- lifecycle information (creation and update dates, vocabulary status, pointers to the people responsible for the conversion and encoding, version information)

Identifiers

Rule 7 (new) Assign unique identifiers for the vocabulary and all the items in the vocabulary

Determine if identifiers are for concepts or words.

Old Rule 5. Design an identifier scheme and pattern

Accompanying text would be more useful if it was something like:

A standard pattern for web-resolvable (HTTP) IRIs is like this:

Identifier = {domain} + {path} + {token-id}

Where ‘domain’ is the web location for the server, e.g. ‘https://geosciml.org/’, ‘http://purl.obolibrary.org/’, ‘https://vocabularies.cessda.eu/’.

Path is a sequence of constants separated by slash characters (‘/’). The path is determined by the requirements of the vocabulary server and implementation decisions when deploying the vocabulary online (see Rule 10). Examples: ‘vocabulary/’, ‘wiki/’, ‘obo/’, ‘classifier/ics/ischart/’.

Token-id is a local name that must be unique in the scope of the domain and path part of the identifier. These may be opaque or use recognizable words….

Representation

Rule 8 (was Rule 6, revised). Create representations for machines and for people.

Create a semantic-standards based vocabulary representations for machine clients using a standard formal language (SKOS, OWL, RDF) and for human clients using HTML.

Create user-readable web page representation for vocabulary and its content

Deployment

Rule 9. Make the identifiers resolve –

Old Rule 4. Select a domain and service for the web identifiers

- Dereferencing the vocabulary or term IRIs will get resources that should contain attribution information,

Rule 10 (was Rule 8). Register (Publish?) the vocabulary –

Load or register the encoded content in a vocabulary service or semantic repository. This section also discusses other ways to publicize the existence of the vocabulary.
