# Supplementary material for: Ten simple rules for making a vocabulary FAIR
Source: PLoS Comput Biol. 2021 Jun 16;17(6):e1009041. doi: 10.1371/journal.pcbi.1009041 (PMC8238180; doi:10.1371/journal.pcbi.1009041)
Supplement: Attachment — Submitted filename: ResponsesToReviewersTenSimpleRulesMakingVocabularyFAIR.docx [file pcbi.1009041.s003.docx]

Specific responses to reviewers comments on **Ten Simple Rules for making a vocabulary FAIR**

**Prepared - 2021-02-28**

**>Reviewer #1**

>The intention of this paper is a useful contribution to address an important problem for >implementation of the Semantic Web, but I think the text needs some work. Recommendation >is to accept with revisions.

>The pdf file contains many comments. Some others are here, followed by some conceptual >suggestions.

We considered each editorial comment from the PDF and accepted or adapted most of them.

>Comments

>There are various requirements that are mixed in with the rules. I think the rules could be >refactored to better encapsulate the requirements (see suggestion in the conceptual section, >below).

We refactored the rules in response to reviewer #1’s suggestions. The revised set of rules do not correspond exactly to reviewer #1’s proposal. The details are described in the conceptual section below.

>The text uses a some terminology that might not be clear to users, e.g.

>annotation (e.g. line 27, 62 80) "An annotation is extra information associated with a particular >point in a document or other piece of information." (https://en.wikipedia.org/wiki/Annotation). >maybe -- annotate: "specify the kinds of entities in data or metadata schema, or the values of >categorical properties in data or metadata"

We clarified the role of terms (vocabulary items) in the context of data (see line 48 - ‘particularly in metadata, column headings, and for some values in datasets’) and have removed most mentions of ‘annotation’ where it was unclearr.

>code e.g. (line 61)

>IRI—in general I think this could be replaced by ‘identifier’, ‘web identifier’, or ‘resolvable >identifier’ most places its used.

We have replaced IRI by ‘resolvable identifier’ and  ‘web identifier’ where appropriate, and used it only after explaining it in the context of ‘Linked Data’

The text assumes familiarity with web architecture. Is it clear that the readers will have this background? Examples: 'web resource' (line 56), web-standard (line 76), 'on the web' line 95, 'web identifier' line 96. If not there should be some background on web architecture and semantic web, at least as a link to a quick overview, covering topics like these:

• URI is a name designed to be unique, there are many schemes. A URI is a name that can be encoded using standard Latin alphabet, numbers and a limited set of other characters (the ASCII character set), following rules specified in IETF RFC (https://tools.ietf.org/html/rfc3986)

• IRI is a generalized URI that allows names constructed with non-Latin characters and a broader set of symbols (anything that can be encoded using UTF-8)

• The World Wide Web (the Web) is a technology for associating identifiers with locations in a global network of computers (the Internet) and retrieving files (resources) from those locations. Retrieved resources can contain resolvable identifiers for other resources, thus creating a web of linked documents.

• A resolvable identifier is an identifier for which there is a known procedure to determine what it identifies and how to obtain a representation of that thing. (‘resolve’ might be less alien to users that ‘dereference’)

• An HTTP URI (or IRI) is a specific kind of identifier that is resolvable using the World Wide Web technology.

• On the Web, an identified thing is a resource; that resource might have multiple representations. A representation is an artifact (digital document on the web) intended to convey information about some resource(a thing);

We have introduced some brief explanations about Linked Data and the semantic web stack. However, we do not believe it is feasible to provide a full introduction to the web-architecture in the context of this paper. Furthermore, while the target audience certainly needs some appreciation of the web-architecture, this is no longer a new thing and there are many available precedents. We expect the reader to rely on the references we have included to get a deeper understanding of these topics, when needed.

Comments on table following line 203

• "Encode each vocabulary term as an owl:Class, or as an instance of an owl:Class if it is the most specific concept in the vocabulary [16]. The term identifier should be assigned as discussed in Rule 5"

• problematic-- why are leafs instances? seems inconsistent, terms in vocab should all be instances or all be classes. Need examples

Removed. The treatment of OWL representations has been simplified to largely match the SKOS expressivity. We indicate that we present only the basic steps for an OWL representation in this paper.

• Can't use equivalent class for synonyms if term is an instance.

Removed.

• "Synonyms can be encoded as annotations" ? examples? what annotation vocabulary?

Specific vocabularies for term metadata and relations are recommended.

• "Add codes and symbols" have to explain what you mean by 'code'

Meaning of ‘code or symbol’ clarified.

• Multiple rdfs:label in owl is tricky (unless different languages...)-- client randomly picks one?

Indeed, we intended to simplify the OWL representation. We have now referred to the SKOS terms for synonyms. We will leave details of a more expressive representation for a future paper…

• Define the hierarchy of terms: in owl, carefully evaluate nature of hierarchy, is it kindOf (use subclass), or partOf, ..

Clarified that sub-class should only be used for ‘is-a-kind-of’ hierarchies.

Conceptual suggestions

A clearer statement of what FAIR means for a vocabulary would be useful. Something like:

FAIR in the context of a vocabulary:

• Find -- search for term and find representation that binds a text label (symbol) with intended meaning (via a text definition), and a resolvable identifier; given an identifier, get a representation to explain what it means; provide metadata documenting provenance.

• accessible-- there is an online mechanism by which given the identifier, a useful representation can be obtained that conveys the meaning and provenance of a vocabulary item; the entire vocabulary can be accessed as a unit online.

• interoperable-- at least one accessible representation conforms to a recognized scheme for serializing information about the vocabulary item

• reusable-- license; sufficient metadata to trust; definitions of terms are sufficient to understand what they mean.

We added a colourful table (sidebar) which summarizes these points (slightly modified with respect to what the reviewer wrote)

Assumptions-- necessary condition for a vocabulary is a set of unique labels. Depending on the nature of the vocabulary, those labels might or might not be bound to unique meaning. There should be a recommendation to specify if identifire is bound to the meaning, or to the label.

Rule 3 - minimum vocabulary content - has been clarified to require unique labels and to mention risks around overlapping or ambiguous definitions. However, we already mentioned the need to encode the vocabulary *as-is* and separate the revision or remediation process from the FAIRification phase, so we do not think it is helpful to make the content check any stronger than stated.

Possible refactoring of rules:

**Vocabulary management and stewardship**

Rule 1. Verify that the legacy-vocabulary license allows repurposing

Rule 2. Determine the governance arrangements and custodian responsible for the legacy vocabulary

Rule 3 (new) Determine the license that will be assigned to the online vocabulary

Rule 4 (was Rule 10). Implement a process for maintaining the vocabulary content and online access

engage with the community for whom the vocabulary is provided, through the subject matter experts that created the vocabulary

**Vocabulary content**

Rule 5 (was Rule 3). Check minimal term definition completeness

• Check that labels are unique

• Have reconcile this with the

Rule 6 (was Rule 7). Add rich metadata –

• Define the scope and purpose of the vocabulary

• provenance and ownership information (citation of or links to the source, pointers to the organization or community responsible for the content),

• lifecycle information (creation and update dates, vocabulary status, pointers to the people responsible for the conversion and encoding, version information)

**Identifiers**

Rule 7 (new) Assign unique identifiers for the vocabulary and all the items in the vocabulary

Determine if identifiers are for concepts or words.

Old Rule 5. Design an identifier scheme and pattern

Accompanying text would be more useful if it was something like:

A standard pattern for web-resolvable (HTTP) IRIs is like this:

Identifier = {domain} + {path} + {token-id}

Where ‘domain’ is the web location for the server, e.g. ‘https://geosciml.org/’, ‘http://purl.obolibrary.org/’, ‘https://vocabularies.cessda.eu/’.

Path is a sequence of constants separated by slash characters (‘/’). The path is determined by the requirements of the vocabulary server and implementation decisions when deploying the vocabulary online (see Rule 10). Examples: ‘vocabulary/’, ‘wiki/’, ‘obo/’, ‘classifier/ics/ischart/’.

Token-id is a local name that must be unique in the scope of the domain and path part of the identifier. These may be opaque or use recognizable words….

**Representation**

Rule 8 (was Rule 6, revised). Create representations for machines and for people.

Create a semantic-standards based vocabulary representations for machine clients using a standard formal language (SKOS, OWL, RDF) and for human clients using HTML.

Create user-readable web page representation for vocabulary and its content

**Deployment**

Rule 9. Make the identifiers resolve –

Old Rule 4. Select a domain and service for the web identifiers

• Dereferencing the vocabulary or term IRIs will get resources that should contain attribution information,

Rule 10 (was Rule 8). Register (Publish?) the vocabulary –

Load or register the encoded content in a vocabulary service or semantic repository. This section also discusses other ways to publicize the existence of the vocabulary.

We refactored the rules, prompted by the suggestion from reviewer #1. However, we have retained our overall organization of the rules in approximately the order they need to be considered and executed, rather than as proposed by reviewer #1 which is more thematic. We want to keep our approach very practical and provide guidance on the steps towards FAIR vocabularies implementation, rather than focus on the conceptual aspects only. The revised sequence is as follows:

|  | **Original submission** | **Reviewer #1 suggestion** | **Revised submission** |
| --- | --- | --- | --- |
| 1 | Verify that the legacy-vocabulary license allows repurposing | 1→1 Verify that the legacy-vocabulary license allows repurposing | 2→1 Determine the governance arrangements and custodian of the legacy vocabulary |
| 2 | Determine the governance arrangements and custodian responsible for the legacy vocabulary | 2→2 Determine the governance arrangements and custodian responsible for the legacy vocabulary | 1,7’→2 Verify that the legacy-vocabulary license allows repurposing, and agree on the license for the FAIR vocabulary |
| 3 | Check minimal term definition completeness | 7→3 Determine the license that will be assigned to the online vocabulary | 3→3 Check term and definition completeness and consistency in the legacy vocabulary |
| 4 | Select a domain and service for the web identifiers | 10→4 Implement a process for maintaining the vocabulary content and online access | 10’→4 Establish a technical maintenance environment for the FAIR vocabulary artefacts |
| 5 | Design an identifier scheme and pattern | 3→5 Check minimal term definition completeness | 4’,5→5 Assign a unique identifier to (a) the vocabulary and (b) each term in the vocabulary |
| 6 | Create a semantic-standards based vocabulary***Interoperability*** | 7→6 Add rich metadata | 6→6 Create machine readable representations of the vocabulary terms |
| 7 | Add rich metadata - ***Reusability*** | 4,5→7 Assign unique identifiers for the vocabulary and all the items in the vocabulary | 7’→7 Add vocabulary metadata |
| 8 | Register the vocabulary - ***Findability*** | 6→8 Create representations for machines and for people | 8→8 Register the vocabulary |
| 9 | Make the IRIs resolve - ***Accessibility*** | 9→9 Make the identifiers resolve | 4’,9→9 Make the vocabulary accessible for humans and machines |
| 10 | Implement a process for maintaining the FAIR vocabulary | 8→10 Register (Publish?) the vocabulary | 10’→10 Implement a process for maintaining the FAIR vocabulary |

**Reviewer #2:** The authors present here a succinct and comprehensive set of advise for both encoding vocabularies in Semantic Web standards and for making vocabularies fit well into the FAIR principles for Data Management. Having been responsible for publishing a number of controlled vocabularies, I find their steps here to a well laid out Best Practice. As such this is a timely paper and the authors are to be congratulated for their input and presentation. I have only one very minor comment, which is that on lines 42, 94 and 95 the render the phrase "linked data" and "linked-data", which I would more normally expect to see as "Linked Data".

Once again - thank you for the opportunity to review this well written paper.

We have changed the text to always refer to “Linked Data”.

**Reviewer #3:**

This paper proposes a set of 10 rules to provide FAIR vocabularies. The work is interesting and worth being published as FAIR data and related initiatives are gaining momentum. In this sense, the first recommendation for improving the paper would be to align the proposed rules to the previous works as "Best Practices for Implementing FAIR Vocabularies and Ontologies on the Web" (<http://ebooks.iospress.nl/volumearticle/56005> and arxiv version<https://arxiv.org/abs/2003.13084>) and the FAIRsFAIR D2.2 FAIR Semantics: First recommendations (<https://zenodo.org/record/3707985>). Authors may also be interested in the special issue<https://www.mitpressjournals.org/doi/full/10.1162/dint_e_00023>

We added a section after the presentation of the rules, including a table in which we align and compare our Ten Simple Rules with elements from three related contributions - the two papers mentioned by this reviewer, plus the W3C Best Practice on Data on the Web.

Other important comments to enhance the current paper are:

Line 171: What do you mean by "Resource Description Framework (RDF) vocabularies"? the references mix the RDF and RDFS recommendations with Dublin Core. RDF is the data model for the web, RDFS is the language for vocabularies and OWL is a language for ontologies. Finally Dublin Core is a controlled vocabulary that happens to be implemented in RDFS and in OWL (depending elements or terms.) Please clarify these concepts. It would be advisable also to clarify in general than OWL is the ontology language and SKOS is an OWL ontology to represent thesaurus, not a particular language itself as in general lines from 175 to 187 might be a bit misleading, in particular lines 185-187 as the constructs that are "missed" in OWL are actually built in SKOS based on OWL and the reasoning features are defined in OWL.

The text has been revised to clarify the role of OWL vs SKOS, and the OWL pattern is simplified to essentially match the SKOS model (since it would be impossible to fully explore OWL patterns in a compact form in this paper). Mentions of ‘elements from standard vocabularies’ have been modified to avoid incorrect implications about their RDF/RDFS/OWL significance.

Regarding the table for steps for SKOS and OWL conversion I would suggest to keep it just for SKOS as there are several points in the OWL column. In general I feel that that column could lead to conceptual errors to the readers, overall not experts in knowledge representation, as it seems to oversimplify a great amount of work about ontological engineering. While the steps for converting to a SKOS are clear and could be automated, for building an ontology there are a number of consideration that should be made and given the type of publication it would be more suitable to keep it to SKOS rather than entering in an ontological engineering work. The points about the table are:

We have retained the OWL column, but simplified it and removed mentions of most advanced topics. The expressivity of the simplified OWL recipe is now essentially matched to SKOS. But we think it is necessary to include OWL in this rule, because this piece of the semantic technology stack is an important part of the pathway to more advanced axiomatization. We have clarified the relationships between SKOS and OWL, and provided additional references that describe the relationship between them.

-- "Identify terms": "or as an instance of an owl:Class if it is the most specific concept in the vocabulary " --> not always, one may want to have all as classes and create individual for the last level. It depends on the use.

Removed.

-- "Encode term labels and synonyms" --> In general, it is a bad practice to use the owl:equivalentClass construct to define synonyms, they could be added as several labels to a given class or with other annotations but not creating N classes in the same ontology unless there is a good reason for that.

We agree that the owl:equivalentClass construct should not be the first option when encoding synonymy and we have now removed its mention. We have rewritten the recommendation to point skos:prefLabel and skos:altLabel for synomyms when relevant.

-- "Define the hierarchy of terms" --> transforming broader/narrower relationships to rdfs:subClassOf would lead to semantic errors and reasoning issues. See that in a thesaurus we can have PC - narrower - mouse. If we make mouse subclass of PC is a semantic inconsistency as individuals of mouse will be classified as individuals of PC. In this cases a transformation is not that simple and if one wants to transform a thesaurus into an ontology there should be some modelling decisions to be taken.

Clarified the kind of relationships that match ‘sub-class’.

-- minor: rdfs:comment is used in steps 3 and 5 for different purposes, the original type of annotation would be lost.

Indeed. We have also suggested using dct:description for textual definition, in order to differentiate it from rdfs:comment.

-- "Add per-item metadata": for the case of ontologies the LOV, MOD<https://github.com/sifrproject/MOD-Ontology> and Widoco guidelines could be included but as said, I would suggest keeping the table for SKOS.

These metadata vocabularies are mentioned in Rule 7 (whole of vocabulary metadata).

Other comments are:

In rule 8 LOV should be added as registry as it is widely used by many communities and Ontobee.

Added.

Line 32 and 61: the term glossary seems to be used as synonym for controlled vocabularies and could be a bit inaccurate. I'd suggest using and referring to the ontology spectrum by McGuinness<http://www.ksl.stanford.edu/people/dlm/papers/ontologies-come-of-age-mit-press-(with-citation).htm>

The material about different kinds of vocabularies has been moved together, with the reference to McGuiness. The use of ‘controlled vocabulary’ is explicitly reserved for governance and maintenance aspects.

Line 61 to 65: I don't see why those example "may not be initially recognised as vocabularies" if word-lists are vocabularies list of terms which are unit of measure are too.

Reworded to clarify the intent, as part of the ontology spectrum discussion.

Minor comments:

Line 73 to 76: the list of digital documents is too narrow, it might include relational databases, XML files, etc.

Yes, additional types of digital but unFAIR resources added.

Line 81: ".." remove one "."

Done.
