# Supplementary material for: Ten simple rules for making a vocabulary FAIR
Source: PLoS Comput Biol. 2021 Jun 16;17(6):e1009041. doi: 10.1371/journal.pcbi.1009041 (PMC8238180; doi:10.1371/journal.pcbi.1009041)
Supplement: Attachment — Submitted filename: Ten simple rules #1 Response to second review.docx [file pcbi.1009041.s004.docx]

Detailed response to second review

Reviewer #1: The revised version is excellent. Thanks for putting this together!

Reviewer #2: This revision looks excellent - I recommend for publishing

Reviewer #3: Thank you very much for your detailed comments and the effort improving the paper.

There are substantial changes in the manuscript addressing the reviewer's comments. I think the paper has improved and actually it a good starting point for converting legacy terminologies to FAIR vocabularies.

I only have some small comments:

Line 73: is there an "and" or "or" missing before "(b)"?

**‘and’ inserted**

Lines 226 to 229: It is common to use # for small vocabularies or ontologies, it would be better to have that mentioned. I understand it is different if you are transforming a huge KOS.

**the paragraph has been revised to reflect the fact that # URIs are OK for small vocabularies**

Table skos and owl, point "Define the hierarchy of terms" in the owl column: "A narrower concept or subclass" --> I would avoid mentioning here "narrower", maybe "A more specific concept or subclass... ". Also, I would add a clarification stating that in owl the subclass is oriented to subsets of elements which is not exactly the same as "narrower/broader" for SKOS.

**the instruction in the OWL column has been revised to clarify that rdf:subClassOf has more precise semantics than skos:broader**

Table skos and owl, point "Define the whole vocabulary" about the sentence: "Every member term should have a rdfs:isDefinedBy relationship to the ontology" --> Could this be optional for when the element URI is defined in the same namespace as the ontology URI?

**the instruction has been revised to make rdfs:isDefinedBy optional for terms in the same namespace**

Regarding the options to transform vocabularies to SKOS or OWL, I find very useful OpenRefine. Not sure how easy to use is for non-experts in RDF or semantic technologies.

**OpenRefine has been added to the list of tools**

**We have also made the following additional small changes, in response to an additional review from a colleague that was received:**

- **Rules 4 and 10 have been clarified to make it clear that Rule 4 describes a technical platform, while Rule 10 relates to a workflow or process**
- **A couple of sentences have been added to the Summary and Conclusions, which reflect back to the motivation from the Introduction**
